# The Attack on President McKinley

**Published:** 1901-09-14

**Authors:** 


### THE ATTACK ON PRESIDENT McKINLEY.

The dastardly attempt on the life of President McKinley, while it has elicited an indignant storm of execration against the band of assassins who dub themselves Anarchists—as if a mere name or creed could lessen the crime of murder—has raised a feeling of sympathy with the wounded President and his wife, which has swept as a great wave around the globe. From the purely surgical standpoint the wound which his assailant inflicted on the President presents points of very considerable interest, for the injury was typically one of those which war experience, both in South Africa and in Cuba, has shown to be very fatal, and yet according to the latest news (we write this on Wednesday evening) the President is doing well. It appears that the bullet which did the mischief—the second bullet—penetrated the abdomen five inches below the left nipple and one-and-a-half inches to the left of the median line, and that on the abdomen being opened, through the line of the bullet wound, it was found to have penetrated the stomach. The opening in the front wall of the stomach was carefully closed with silk sutures, after which a search was made for an opening in the opposite wall. This was found and was also closed in the same way. No injury to the intestines or other abdominal organ was discovered. The bullet, which was not found, is probably lodged in the muscles behind.

A wound like this, a penetrating gun-shot wound above the umbilicus, is one of the most fatal of abdominal injuries. The question of treatment is a matter of the most urgent importance, and the American surgeons are to be congratulated on the promptitude with which they proceeded to operation, for a good deal has happened of late to tempt men to let such cases alone. At the beginning of the war in South Africa everyone was for operating in cases of penetrating wounds of the abdomen. Experience gained in civil life had demonstrated how successfully operations on the abdomen might be performed, while previous wars had shown how desperately fatal the let-alone treatment had been. Thus, in the early stages of the war, many gun-shot wounds of the abdomen were treated by operation, the abdomen being opened and the internal wounds sought for and sewn up. Even in the most experienced hands, however, the results were almost uniformly disappointing. The causes for these disastrous results are not far to seek, and have been well described by Sir Frederick Treves—delay and difficulties of transport, jolting and disturbance, and the impossibility of securing complete asepsis. On the other hand experience soon showed that a very considerable proportion of the cases which were let alone did well, especially those in which the wound was situated below the umbilicus. Thus a revulsion took place against operating in gun-shot wounds of the abdomen. But this case of the President should serve to remind us that difficult and often impossible of practical application as surgical principles may be in a field hospital after a great battle, the principles remain true when the details can be properly carried out. Fortunately the organisers of the great show at Buffalo had provided an admirable little emergency hospital within the grounds, and to this without delay the wounded President was removed; fortunately, again, skilful surgeons were obtainable, also without delay; and the result has been so far most satisfactory. Let us hope it will continue.

